# FAMILY TIES AND HEALTH CARE DECISION-MAKING IN LATE LIFE: EXAMINING FINANCIAL DEPENDENCY IN HEALTH FOR INDIA

**DOI:** 10.1093/geroni/igae098.3091

**Published:** 2024-12-31

**Authors:** Atul Pandey, Isha Sharma

**Affiliations:** Indian Institute of Technology Jodhpur, Jodhpur, Rajasthan, India; Indian Institute of Technology Jodhpur, Jodhpur, Rajasthan, India

## Abstract

With the increasing number of age 60 years and above population, it is becoming important to improve healthcare and economic assurance for grandparents/parents. Family ties and culture is a way of consideration of healthcare choices and providing financial support. Family member’s financial stability assure the finance for healthcare for aged population. The dissolution of traditional extended families in India is closely related to the above-described issues, which also lead to rising economic inequality, urbanization, modernization, growing individualism, and a greater emphasis on healthcare choices. The rise in nuclear families and longer life expectancies has led to an increase in the cost of caring for the late ageing population. This study is examining the role of family ties and healthcare decision making through the lens of financial dependency. This study uses Longitudinal Ageing Study in India (LASI) i.e. secondary data source capturing 31464 respondents with the age of 60 years and above. It again classified into the male (16366) and female (15098) cohort. The data provide significant information related to the coverage of financial security. One out of every five individuals with age 60 years and above is enrolled in an organized sector’s pension plan. A sizable share (20.65%) of the population is covered by central and state pension programs. In contrast, only a small percentage of India’s retired population (2.1%) is covered by private pension, or employer-funded pension schemes. Just 7% of individuals believe their family’s financial situation is adequate, whereas 53% comes from middle class family.

